# Commissioning of a 3D image‐based treatment planning system for high‐dose‐rate brachytherapy of cervical cancer

**DOI:** 10.1120/jacmp.v17i2.5818

**Published:** 2016-03-08

**Authors:** Yongbok Kim, Joseph M. Modrick, Edward C. Pennington, Yusung Kim

**Affiliations:** ^1^ Department of Radiation Oncology College of Medicine The University of Arizona Tucson AZ; ^2^ Department of Radiation Oncology Carver College of Medicine The University of Iowa Iowa City IA USA

**Keywords:** software commissioning, 3D image‐based, treatment planning system, volume optimization, high‐dose‐rate brachytherapy, cervical cancer

## Abstract

The objective of this work is to present commissioning procedures to clinically implement a three‐dimensional (3D), image‐based, treatment‐planning system (TPS) for high‐dose‐rate (HDR) brachytherapy (BT) for gynecological (GYN) cancer. The physical dimensions of the GYN applicators and their values in the virtual applicator library were varied by 0.4 mm of their nominal values. Reconstruction uncertainties of the titanium tandem and ovoids (T&O) were less than 0.4 mm on CT phantom studies and on average between 0.8‐1.0 mm on MRI when compared with X‐rays. In‐house software, HDRCalculator, was developed to check HDR plan parameters such as independently verifying active tandem or cylinder probe length and ovoid or cylinder size, source calibration and treatment date, and differences between average Point A dose and prescription dose. Dose‐volume histograms were validated using another independent TPS. Comprehensive procedures to commission volume optimization algorithms and process in 3D image‐based planning were presented. For the difference between line and volume optimizations, the average absolute differences as a percentage were 1.4% for total reference air KERMA (TRAK) and 1.1% for Point A dose. Volume optimization consistency tests between versions resulted in average absolute differences in 0.2% for TRAK and 0.9 s (0.2%) for total treatment time. The data revealed that the optimizer should run for at least 1 min in order to avoid more than 0.6% dwell time changes. For clinical GYN T&O cases, three different volume optimization techniques (graphical optimization, pure inverse planning, and hybrid inverse optimization) were investigated by comparing them against a conventional Point A technique. End‐to‐end testing was performed using a T&O phantom to ensure no errors or inconsistencies occurred from imaging through to planning and delivery. The proposed commissioning procedures provide a clinically safe implementation technique for 3D image‐based TPS for HDR BT for GYN cancer.

PACS number(s): 87.55.D‐

## I. INTRODUCTION

The use of computed tomography (CT) imaging for high‐dose‐rate (HDR) brachytherapy (BT) treatment planning is increasing, particularly for the management of gynecological (GYN) malignancies. The most recent comprehensive survey data in 2007 showed that 70% of patients in the United States treated using GYN BT procedures had a CT scan. Traditionally, a reference point called “Point A” was selected to receive the prescribed dose using two‐dimensional (2D) film‐based treatment planning. However, in the 2007 study, 3D CT image‐based treatment planning was used most (55%) followed by 2D film‐based planning (43%), while magnetic resonance image (MRI)‐based planning enjoyed limited use (2%).^(1)^ To safely implement 3D image‐based HDR BT for cervical cancer, the European GYN group, Groupe Européen de Curiethérapie (GEC)—European Society for Radiotherapy and Oncology (ESTRO) working group provided several valuable guidelines for 3D image‐based HDR planning. These guidelines focused on target delineation in MRI;^2^, ^3^ dosimetry, physics tests, and radiation biology;^(4)^ commissioning and applicator reconstruction for plastic applicators;^(5)^ and MRI protocol recommendations for better image acquisition.^(6)^ In addition, the challenges implicit in applicator reconstruction uncertainty were investigated for CT image‐based HDR planning^(7)^ and for 0.3 Tesla (T) open MR scanner planning.^(8)^ However, note that the reconstruction accuracy validations for either titanium applicators or high resolution 3T MRI are very limited. The dosimetric impact from reconstruction uncertainty was also evaluated.^9^, ^10^ In the United States, the American Brachytherapy Society (ABS) proposed guide lines^11^, ^12^ and consensus^(13)^ was found for 3D image‐based intracavitary BT for cervical cancer.

As far as the quality assurance (QA) program is concerned, the American Association of Physicists in Medicine (AAPM) has historically provided guidelines relating to HDR BT. Task Group (TG) 41^(14)^ (published in 1993) described afterloader technology; TG 56^(15)^ (1997) presented a code of practice for BT physics including QA for HDR units and HDR treatment planning and evaluation; TG 59^(16)^ (1998) focused on HDR treatment delivery to assure the safe delivery of HDR treatments. AAPM TG 59 distinguished between systematic errors and random errors in HDR procedures. In addition, TG 40^(17)^ (1993) discussed comprehensive QA guidelines for radiation oncology including BT treatment planning QA. Although TG 53^(18)^ (1998) described QA for clinical radiotherapy treatment planning focusing, mainly on external beam radiation therapy, it also included recommendations for the commissioning of BT dose calculations. However, all these TG reports addressed technical issues regarding 2D image‐based BT procedures only. While these TG guidelines still provide a detailed conceptual framework for use in the era of modern 3D image‐guided BT, they have been eclipsed by the technical advancements used in 3D image guidance, 3D image‐guided planning, delivery, and verification in HDR BT.^(19)^ A practical guide to BT quality control equipment including treatment planning systems (TPS) QA was documented in 2004 by ESTRO as the European standard for QA.^(20)^ However, this guide does not include recent advances related to the complexity of 3D image‐guided treatment planning for GYN BT.

This study presents a detailed explanation of the physics required for the commissioning of 3D image‐based TPS using HDR BT for the treatment of cervical cancer by adapting concepts from the literature^2^, ^3^, ^4^, ^5^, ^6^, ^7^, ^8^, ^9^, ^15^, ^18^, ^20^, ^21^ and focuses mainly on the 3D‐image based HDR BT planning challenges that are not fully addressed in the literature. These include: the applicator library in the TPS, titanium applicator reconstruction accuracy on MRI exclusively used for treatment planning, image manipulation tools in 3D image based TPS, dose calculation algorithm, and dose‐volume histogram (DVH). Furthermore, volume optimization algorithms in 3D image‐based planning were comprehensively investigated with respect to the difference between line and volume optimizations, volume optimization consistency between different versions and its sensitivity to different optimization times, as well as a comparison of different volumetric optimization techniques for clinical GYN tandem‐and‐ovoids (T&O) cases. Finally, end‐to‐end testing is presented. In this study, a commercial TPS (BrachyVision, version 6.1, Varian Medical Systems, Inc. Palo Alto, CA) was upgraded to higher versions 6.5, 8.5, 8.9 and 10.0 as the study proceeded.

## II. MATERIALS AND METHODS

### A. Virtual brachytherapy applicator library

The dimensions of the physical BT applicator sets were compared to those in the corresponding virtual applicator library in the TPS (SolidApplicator, BrachyVision, Varian Medical Systems, Inc.), to ensure compatibility with, and reconstruction accuracy of, the applicators on CT and MR images. To demonstrate the clinical procedures for the commissioning of BT applicators and validate their reconstruction accuracy on 3D image‐based TPS, the results of the following two applicators are presented: a titanium Fletcher‐Suit‐Declos (FSD) T&O, and single‐channel vaginal cylinder (VC). A titanium T&O set is CT‐compatible and MR‐conditional, while the stainless steel probe of VC is only CT‐compatible. To mimic clinical cases, we followed a GYN HDR‐BT CT scan protocol presented in a previous study,^(22)^ and acquired all scans using a Siemens Biograph 40 PET/CT scanner (Siemens Medical Systems, Erlangen, Germany). A CT scan was taken with a 0.6 mm collimator and reconstructed with both 1 mm and 3 mm slices. The 1 mm slices were used for applicator reconstruction, while those of the 3 mm slice thickness were used for physician contouring. The details of the MRI acquisition protocols are described in a previous study.^(23)^ A previously developed in‐house HDR QA phantom was used in this study (See the Figure 1 in Kim et al.^(23)^). The HDR QA phantom was developed to suspend an applicator and to provide a reference for quantifying the image tools in 3D image‐based TPS.^(23)^

**Figure 1:**
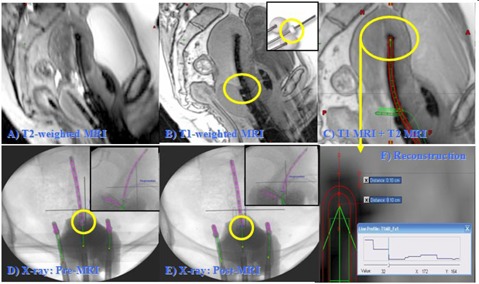
Reconstruction of titanium T&O applicator on MR images: (a) T2‐weighted MRI with 3 mm slice thickness; (b) T1‐weighted MRI with clear flange information; (c) fused MRI (T1‐ and T2‐weighted); (d) X‐ray prior to MR scan; (e) X‐ray following MR scan; (f) tandem tip of reconstructed applicator is described in detail on MR images with MR intensity profile. The flange position in (d) and (e) enables to calculate Δd. From the maximum intensity change point, we account for 1 mm artifacts and another 1 mm for catheter thickness.

### B. Titanium T&O applicator reconstruction accuracy on MRI‐only treatment planning

Three Tesla (3T) MRI‐guided, titanium T&O HDR BT volume optimization planning is institutional standard care.^23^, ^24^, ^25^, ^26^ We validated the artifacts and distortions of titanium in the high 3T magnetic field^(23)^ in order to use the 3T MRI as a single planning imaging modality for MRI‐guided GYN BT. The titanium T&O applicator reconstruction accuracy on 3T MRI was validated by comparing MR image‐based plans with conventional X‐ray‐based plans for 19 randomly selected clinical T&O plans (Fig. 1). The titanium T&O applicator was exclusively reconstructed on T1‐weighted MR images (see Fig. 1(b)) from the applicator library that is shown in Figs. 1(c) and (f). For comparison, two T&O reconstructions were performed based on orthogonal radiographs before and after the MR scan (pre‐ and post‐X‐ray) as seen in Figs. 1(d) and (e). The measurement of vector distances “d” were performed between each T&O dwell position and a cervical marker (flange) evident in the circles on Figs. 1(b), (d), and (e). The difference in distance (Δd) between the MR images and the pre‐X‐ray $\left(d_{\text{preX}-\text{ray}}-d_{\text{MRI}}\right)$ and between the MRI and post‐X‐ray $\left(d_{\text{MRI}}-d_{\text{postX}-\text{ray}}\right)$ were measured. The Δd values were used to quantify the reconstruction uncertainty of HDR planning conducted exclusively using MR imaging. The average ± standard deviation (SD) of Δd for each dwell position was determined for each T&O plan.

### C. Imaging tools in 3D image‐based TPS

The multiple known reference dimensions of the in‐house HDR QA phantom (see Fig. 1 in Kim et al.^(23)^) were used to validate the accuracy of the distance measurement tool in the 3D image‐based TPS. The manual distance measurement performed on CT and MR images imported from the scanner to the 3D image‐based TPS was repeated 2 or 3 times to ensure consistency and establish an average value from the measurements reported. The spatial resolution of the CT images was 0.3 mm by 0.3 mm with a 1 mm slice thickness. The spatial resolution of female pelvic 3T MR images was 1.2 mm by 0.9 mm and 1.0 mm by 1.0 mm for T1‐weighted gradient‐echo and T2‐weighted turbo‐spin‐echo protocols, respectively.^(23)^ The scaling, translation and rotation tools in the 3D image‐based TPS were validated using the T&O applicator. The applicator was manually magnified, shrunk, translated, and rotated and scaling was validated by visual inspection. The translation and rotation of the entire applicator was verified by comparing the doses to 40 reference points in the modified plan after the applicator was translated/rotated to those in the original plan. These points are: point A left, point A right, The International Commission on Radiation Units and Measurements (ICRU) report 38 defined bladder and rectum points, five rectal makers, four bladder markers, four left ovoid surface markers, four right ovoid surface marks, and other 19 other reference points that include the ovoid surface points and line optimization points. The tandem length was independently measured in the scaled and rotated images to ensure the consistency of the applicator length. In addition, the dose distribution of the modified T&O plans was visually compared with its original T&O plan.

### D. Dosimetric validations

#### D.1 Dose calculation algorithm

**Figure 2:**
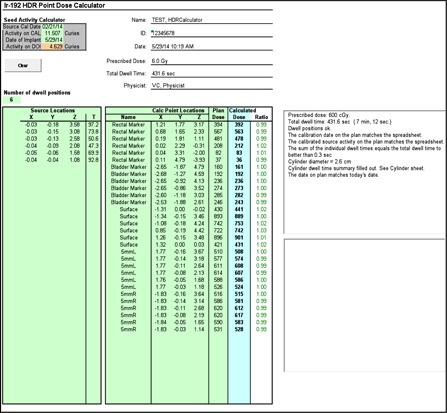
In‐house independent HDR dose calculation and HDR plan quality verification tool compared to the TPS plan for vagina cylinder HDR.

The serial number of a new source wire provided by the vendor was entered into the TPS. The same source model identification “VS Ir‐192 (5 mm)” was used for both old and new versions of the TPS. The parameter of the source center to tip of source wire distance in the device model (VS200 iX, Varian Medical Systems, Inc.) is set to 3.5 mm. General source model properties were compared between versions as follow: source identification, isotope name, manufacturer, half‐life, source type, dose calculation model, dose rate constant, and air kinetic energy released per unit mass (KERMA) strength. Additionally, tables for anisotropy function and radial dose function (scatter function) were compared between source versions and verified. All source data comparisons were printed in hard copy and also made available online as an electronic copy. To verify the source activity of the TPS at a specific treatment date, an independent decay table was generated using the same ^192^Ir source half‐life (73.83 days) as that in the TPS. The AAPM TG 43 and the updated (AAPM TG 43U1) recommendations^27^, ^28^, ^29^ were used as the basis for the dose calculation algorithm in the TPS (BrachyVision, Varian Medical Systems, Inc.). AAPM TG 53 guidelines prescribe that the dose calculation algorithm in the TPS be independently validated with another dose calculation. We developed in‐house software (called HDRCalculator) to verify the dose calculation of the TPS and several aspects of HDR plan quality. It followed the TG 43 parameters prescribed for ^192^Ir in BrachyVision TPS^(30)^ and excluded the anisotropic factor in the TG 43 recommendations. Besides performing dose comparisons, HDRCalculator is capable of checking HDR plan quality such as step size of source positions (5 mm in our clinic) source calibration date, first source position (at 120 cm in our clinic), differences between average Point A dose and prescription dose, treatment date, and the presence of the normalization line of Point A^(13)^ or Point H^(11)^ left and right. It also independently estimates each ovoid size, tandem active source length, and proper definition of ICRU Report #38 defined rectal and bladder point. A HDR plan is exported in a text file and the coordinate information such as each dwell position, the points of optimization lines, the points of ovoid surface, rectal and bladder points, and Point A left and right points. For instance, the active source length is estimated using the coordinates of each dwell position, while each ovoid size is determined by using coordinates of dwell positions and ovoid surface reference lines. Ovoid size is estimated by using the coordinate information of ovoid dwell positions and ovoid surface points. This is in order for a planner to detect errors during treatment planning (Fig. 2).

Additionally, a dose calculation algorithm in the TPS was validated for a simple case where a single dwell position was located at the origin (x=0,y=0, and z=0) with 200 s of dwell time. The new version of the TPS was compared to the previous version by evaluating four parameters of the plans: each dwell time in seconds, ^192^Ir source activity in mCi, total reference air KERMA (TRAK) in cGycm^2^ based upon ICRU Report 38,^(31)^ and total treatment time in seconds.

#### D.2 Dose‐volume histogram

While 2D image‐based treatment planning reports dose to points of interest such as point A or H, 3D image‐based treatment planning is able to generate DVHs based on target volume, organs at risk (OARs) or both, as delineated in the planning 3D images (mostly CT or MRI). This DVH information is used to evaluate a treatment plan from a clinical perspective. Current GEC‐ESTRO recommendations^3^, ^4^, ^32^, ^33^ for GYN HDR BT describe the use of dose‐volume parameters such as D2cc for OAR and D90 for target coverage that more closely correlate with clinical outcomes than the legacy recommendations of maximal doses from ICRU 38.^(31)^

Prior to the clinical use of DVH data from a commercial TPS, the DVH data have to be independently validated with an existing TPS. When the TPS software is upgraded, the DVH data of the new version should be validated against that of the legacy version.

### E. Volume optimization

#### E.1 Optimization algorithm changes in TPS from line optimization only to line and volume optimization

Most current 3D image‐based HDR BT TPSs offer a volume optimization algorithm that uses an inverse planning routine for the optimal dwell time distribution that corresponds to the dwell positions as defined by the HDR applicator(s) as seen on 3D images. The input data for the inverse planning procedures are a set of user‐defined dosimetric constraints on target and OARs surfaces or volumes in order to minimize the difference between desired DVHs and computed DVHs. For a commercial TPS (BrachyVision, version 6.1 or before, Varian Medical Systems, Inc.), the Nelder‐Mead simplex optimization method was used for the line optimization algorithm. In order to offer line and volume optimization, the Nelder‐Mead simplex optimization algorithm was updated for BrachyVision version 6.5 and later. Although there is a study^(34)^ comparing different dose calculations among different TPSs, no studies describe the verification of the optimization algorithm changes. Prior to the clinical use of a 3D volume‐based optimization algorithm in a new 3D image‐based TPS, the algorithm must be validated against the previous version to ensure agreement between the legacy and new versions of the TPS.

A significant change to the inverse planning algorithm used in the BrachyVision TPS was validated when upgrading from a line only optimization (Version 6.1) to a line and volume optimization algorithm (Version 6.5). Ten previous clinical T&O plans were randomly selected for various prescription doses (range 5.5‐7.4 Gy), active tandem lengths (range: 5.5‐7.0 cm), and ovoid sizes (range: 1.6‐2.6 cm) (Table 1). These clinical Point A‐based T&O plans were generated in accordance with American Brachytherapy Society (ABS) recommendations^11^, ^13^ and used the line optimization algorithm available in version 6.1 of the TPS. This algorithm established a set of dosimetric constraints on the four reference lines based upon its prescription dose (two bilateral lines parallel to the tandem and a lateral line parallel to each ovoid, which will be described in detail for Case B in the Materials & Methods section E.2 below). Hereafter, Point A‐based T&O plans refer to the plans generated this way, the details of which are described by Anderson et al.^(35)^ These clinical Point A based T&O plans were exported from the legacy version of the TPS and then imported into the upgraded TPS (version 6.5). Applicator geometry, 3D imaging datasets, and dose constraints for each reference line were kept the same while using different versions of the inverse planning routine. Even though different versions of the software were used (6.1 and 6.5), both plans were identical. Namely the operator‐supplied input data for both plans were identical. Although version 6.5 is capable of volume optimization based on volume information (high risk clinical target volume (HR‐CTV) or OARs) the same optimization based on four virtual lines was used in this test. For each clinical plan, an additional plan using the newer version 6.5 was generated and three parameters were compared between the two plans. They were: TRAK value in cGycm^2^, volume enclosed by the 100% prescription dose $\left({\text{VOL}}_{100\%\text{Rx}}\right)$, and Point A dose.

**Table 1:** Ten clinical T & O cases to compare volume‐based optimization with line‐based optimization. Three parameters were compared between two plans: total reference air KERMA (TRAK), volume receiving 100% prescription dose $\left({\text{VOL}}_{100\%\text{Rx}}\right)$, and Point A dose averaged between left and right points. The values of volume‐based optimized plan were normalized by those of line‐based optimized plan

*Case*	*1*	*2*	*3*	*4*	*5*	*6*	*7*	*8*	*9*	*10*
Prescription dose (Gy)	7	7	7	5.5	7.4	7	7	5.5	5.5	6
Active tandem length (cm)	7	6	6	7	5.5	7	6.5	6.5	6.5	6.5
Ovoid size (cm)	2.5	2	1.6	2	2	2	2	2	2.6	2
*Normalized Values of Three Parameters for Volume‐based Optimized Plan*										
TRAK(cGy&mdot;cm^2^)	1.01	1.00	0.99	0.99	1.02	1.01	1.02	0.99	1.02	1.03
${\text{VOL}}_{100\%\text{Rx}}$	1.13	1.02	0.86	1.01	1.05	1.05	1.05	1.03	1.06	1.07
Average Point A Dose	0.99	0.99	0.98	0.94	1.01	1.00	1.00	1.00	1.00	1.00

#### E.2 Volume optimization consistency between versions

In order to validate the optimization consistency (BrachyVision version 6.5 or higher) in the TPS, two clinical benchmark cases (Case A and B) using different applicator sets were examined using the two different TPS versions. Case Ahas 10 dwell positions to mimic the 5 cm length of a VC HDR plan; Case B is a typical Point A based T&O with 5 cm active length of tandem (10 dwell positions) and 2 cm active length of ovoids (4 dwell positions for each ovoid). Both Case A and B used lines as optimization objectives. For Case A, a 5 cm virtual line was generated laterally 0.5 cm away from the cylinder surface and dwell time distribution was optimized to deliver the prescribed dose to dose points along the virtual line. For Case B, four virtual lines were generated: two 5 cm virtual lines bilaterally 2 cm away from the tandem in parallel; a 2 cm virtual line laterally away from the catheter of each ovoid by 0.5 cm plus radius of ovoid. Volume optimization was performed to deliver the prescribed dose to dose points along the four virtual lines. For this benchmark test, the clinical treatment plans were exported from the legacy version of the TPS and imported into the new version. Hence, the dose distributions obtained in the new version ideally mirrors those of the previous version because all other parameters are the same except for different version of optimization. The same source was used for the same geometry of applicator and optimization parameters for each virtual line were also the same between two versions. Optimization was performed in the new version and the resultant optimized plan was compared to the previous version plan by evaluating the four parameters of the plan: each dwell time, source activity, TRAK, and total treatment time. This test validated the robustness of the volume optimization engine in the upgraded version of the TPS.

#### E.3 Optimization sensitivity to different optimization time

The treatment plan dependency upon the optimization time was established by using five different optimization times: 10 s, 30 s, 1 min, 1.5 min, and 2 min for the typical Point A based T&O plan (clinical benchmark Case B). The 2 min is used as a reference optimization time.

#### E.4 Comparison of three different volume optimization techniques

**Figure 3:**
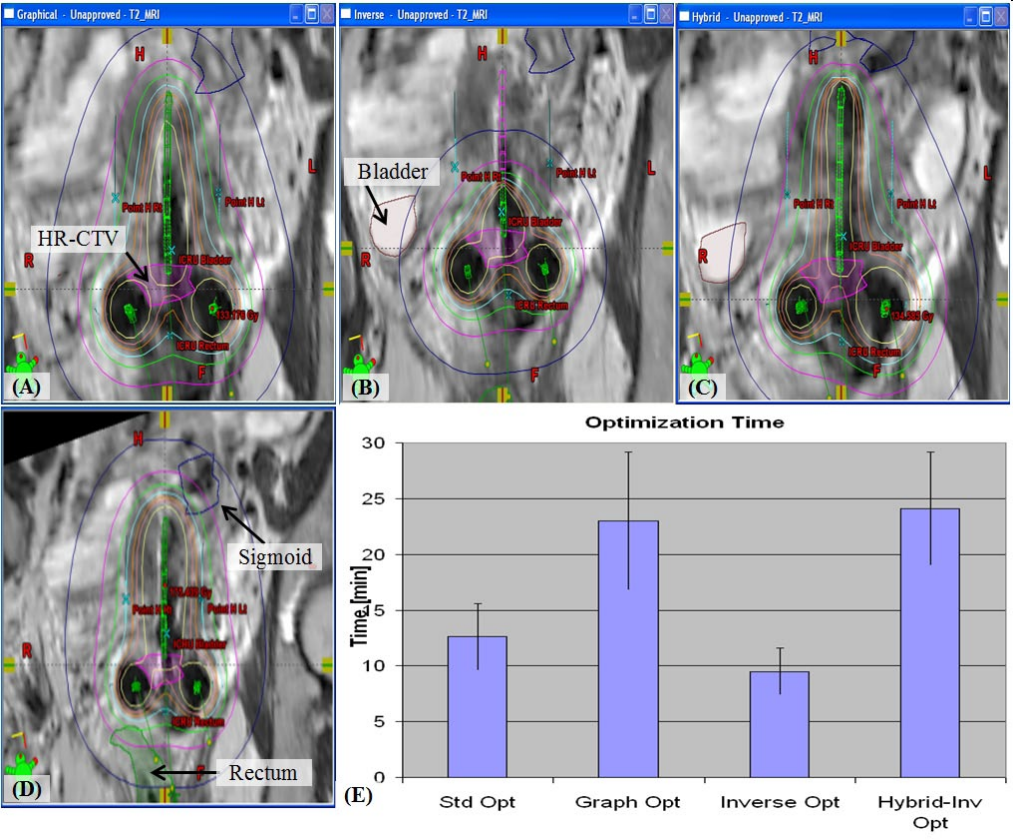
Comparison of dose distributions of different volume optimization techniques of graphical optimization (a), pure inverse optimization (b), and hybrid inverse optimization (c) when compared to that of conventional Point A planning technique (d) for a T&O case. Optimization times spent for each technique are presented (e).

There are three different volume optimization approaches or techniques currently available to generate a conformal brachytherapy plans adapted to the information obtained from 3D images: graphical optimization, pure inverse planning, and hybrid inverse optimization. To evaluate the effectiveness of these techniques, three additional optimization plans were retrospectively generated for a clinical T&O treatment plan. Following ABS guidelines,^13^, ^36^ a pear‐shape dose distribution (Fig. 3(d)) was obtained using four reference optimization lines. It was a conventional Point A plan to be used as a reference plan for the three other optimization plans. It was tested on eight clinical T&O patients (Cases 1‐8). The average ± standard deviation (SD) values of HR‐CTV volume were 37±31 cc for cervical cancers between stages IB1‐IIIB. Graphical optimization refers to a manual graphical dose shaping technique to improve the conventional Point A plan (Fig. 3(a)). The pure inverse planning method uses a set of dose constraints exclusively without any manual adjustment or without generating a Point A plan (see Fig. 3(b)). Target dose constraint was extrapolated from the prescribed dose of 80‐84 Gy in EQD2 (equivalent dose in 2 Gy fraction) and dose constraints on OARs were based on GEC‐ESTRO recommendations:^3^, ^4^, ^5^, ^6^ D2cc<75 Gy in EQD2 for rectum and sigmoid; D2cc<90 Gy in EQD2 for bladder. Prior to initiating the optimization program, all dwell times were set to zero to eliminate any bias. If the optimized dose distribution is not acceptable, dose constraints are readjusted and the optimization process is repeated until a clinically sound plan is created. In contrast, the hybrid inverse optimization plan started as a conventional Point A plan, followed by inverse planning with adaptive dose constraints to improve the DVH parameters of the conventional Point A plan. Finally, the optimized plan was refined with additional dose sculpting using graphical optimization (Fig. 3(c)). These three “replans” using different optimization techniques were evaluated using their DVH parameters and planning time, and compared to the original Point A plan. The dosimetric parameters used were the D90 value for HR‐CTV as target and D2cc for OARs.

### F. End‐to‐end test

As an end‐to‐end test, a dry run test was performed starting with 3D imaging acquisition, 3D image‐based planning, then the HDR delivery using the in‐house HDR QA phantom previously described.

## III. RESULTS

### A. Virtual brachytherapy applicator library

The applicator library was validated by comparing the measured data against the values from the applicator library provided by the vendor. Because CT images are the most geometrically reliable 3D image datasets, the physical dimensions of each applicator were measured on their CT image datasets and then compared to their virtual dimensions in the applicator library (BrachyVision v10.0, Varian Medical Systems, Inc.). Since the tip thickness affects the applicator reconstruction, the physical dimensions of the tip thickness were quantified. The measured data are summarized in Table 2 for the T&O and VC applicators and the nominal values are considered reference data. The average value is a result of at least two measurements. All applicator library and measured values were within 0.4 mm of their nominal values. The uncertainties in the measured values are half millimeter due to its 1 mm slice thickness.

**Table 2:** Measurements of physical tip thickness on CT images and their comparison with nominal values and values in applicator library for T&O and vaginal cylinder (VC) applicator

	*Tandem and Ovoids (T&O)*			*Vaginal Cylinder (VC)*					
	*Tandem*	*Left Ovoid*	*Right Ovoid*	*Probe*	*2.0 cm VC*	*2.3 cm VC*	*2.6 cm VC*	*3.0 cm VC*	*3.5 cm VC*
Nominal thickness (NT) (mm)	0.95	0.95	0.95	0.3	5.4	6.5	7.7	9.2	11.1
Applicator library thickness (ALT) (mm)	0.6	0.9	0.9	0.3	5.3	6.4	7.6	9.2	11.1
Measured thickness (MT) (mm)	1.0	1.2	1.3	0.6	5.5	6.6	7.8	9.4	11.2
Difference: (NT)‐(ALT) (mm)	0.35	0.05	0.05	0.0	0.1	0.1	0.1	0	0
Difference: (NT)‐(MT) (mm)	−0.05	−0.25	−0.35	−0.3	−0.1	−0.1	−0.1	−0.2	−0.1

### B. Titanium T&O applicator reconstruction accuracy on MRI only treatment planning

**Figure 4:**
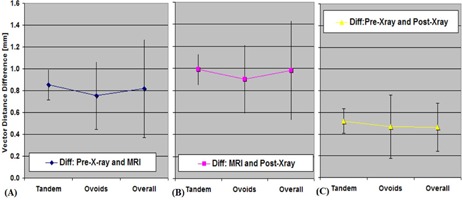
The values of Δd for 19 T&O plans (a) between pre‐X‐ray and MRI; (b) between MRI and post‐X‐ray; (c) between pre‐X‐ray and post‐X‐ray The overall reconstruction uncertainties of T&O on MRI are on average less than 1.1 mm regardless of control datasets (i.e., pre‐ or post‐X‐ray). In addition, panel (c) demonstrates the overall reconstruction uncertainties of T&O between two control X‐ray datasets are on average less than 0.6 mm.

The average ± SD of Δd for each dwell position was recorded for each T&O plan and the results are depicted in Fig. 4. For all T&O dwell positions Δd was on average 0.8±0.5 mm for the $\left(d_{\text{preX}-\text{ray}}-d_{\text{MRI}}\right)$ value (see Fig. 4(a)) and Δd was 10±0.5 mm for the $\left(d_{\text{MRI}}-d_{\text{postX}-\text{ray}}\right)$ value (see Fig. 4(b)). For tandem dwell positions the Δd values were on average 0.9±0.1 mm. For ovoids dwell positions the Δd values were 0.8±0.3 mm for the $\left(d_{\text{preX}-\text{ray}}-d_{\text{MRI}}\right)$ value comparisons. Regarding the value of $\left(d_{\text{MRI}}-d_{\text{postX}-\text{ray}}\right)$ comparisons, the Δd values were on average 1.0±0.2 mm at the tandem dwell positions and 0.9±0.4 mm at the ovoid dwell positions. Two T&O cases (2 out of 19) showed an average Δd>2 mm. For the 19 T&O cases, the largest Δd values were 3.7±0.3 mm and 3.2±0.8 mm at the tandem for the values of $\left(d_{\text{preX}-\text{ray}}-d_{\text{MRI}}\right)$ and $\left(d_{\text{MRI}}-d_{\text{preX}-\text{ray}}\right)$, respectively. When MRI is used exclusively as a 3D image‐based planning dataset, we urge caution with regard to its reconstruction accuracy.

### C. Imaging tools in 3D image‐based TPS

**Figure 5:**
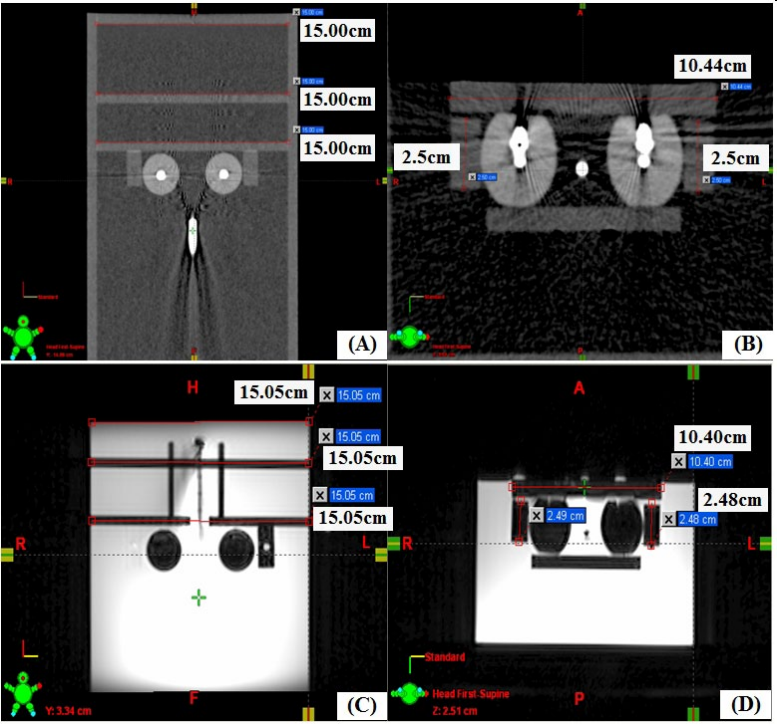
Imported CT ((a) and (b)) and high resolution (3 Tesla) MRI ((c) and (d)) dataset's integrity was checked with known dimensions using QA phantom.

The average discrepancy between the measured distance on the CT images (see Figs. 5(a) and (b)) and the nominal value was 0.65 mm. The discrepancy between the measured distances on the 3T MR images (Figs. 5(c) and (d)) and the nominal value was 0.5 mm. Both CT and MR images presented measurement discrepancies of less than 0.7 mm over the nominal values. If the slice thickness is decreased in the reconstruction of the MR images, both image contrast and spatial resolution are similarly reduced due to the reduction in MR signal.^(23)^

When the scaling, translation, and rotation tools were validated in the 3D image‐based TPS using the T&O applicator, the calculated doses for the 40 dose points in the modified T&O plans were exactly the same as those in the original T&O plan. The measured tandem length was consistently the same for all manipulated images. The dose distribution was identical to that of the original plan despite the manipulation of the applicator in the TPS. A 3D DICOM (Digital Imaging and Communications in Medicine) image data transfer configuration was tested to ensure the connectivity of image transfer capability between CT/MR scanners and the TPS computer. This also ensured the consistency and accuracy of transferred 3D DICOM image data in the TPS compared to the original images.

### D. Dosimetric validations

#### D.1 Dose calculation algorithm

HDRCalculator, our in‐house developed software verified the dose calculation of the TPS and HDR plan quality. Figure 2 compares the calculated doses with plan doses using examples of a vaginal cylinder HDR plan.^(37)^ For 30 dose points in the VC plan, the dose difference was <3%. For the T&O plan (see Appendix A), the dose discrepancy was <3% for the 40 dose points except for the rectal dose point which was defined by ICRU Report 38^(31)^ and located too close to the ^192^Ir source. The dose difference for the rectal dose was 5% because of the high anisotropic factor associated with its location in the ovoid which was ignored in the HDRCalculator.

For the simple case having a single dwell position at the origin (x=0,y=0, and z=0), the four parameters were exactly the same between two different versions of the TPS.

#### D.2 Dose‐volume histogram

**Figure 6:**
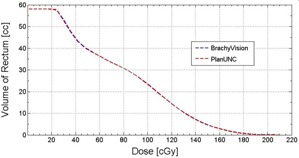
Comparison of rectal dose‐volume histograms between BrachyVision TPS and PlanUNC TPS.

Any open or other commercially obtained TPS may be used, provided it has the capability to import and export plans in DICOM format. In this study, an openly accessible TPS called “PlanUNC” TPS^(38)^ was used to generate a DVH. A clinical T&O treatment plan was exported in DICOM format and imported to the PlanUNC TPS, including the planning CT images, anatomy contours, and the calculated dose matrix. As an example, the rectal DVH was reproduced in the PlanUNC TPS and compared to a commercial HDR BT TPS (BrachyVision v8.5, Varian Medical Systems, Inc.). Figure 6 displays rectal DVHs from the two different TPSs. Both DVHs are so similar that they overlap each other. The dose bin size was 6 cGy and no rectal volume received more than 210 cGy. The average and maximum difference in DVH rectal volumes between the two TPSs is 0.1 cc and 0.5 cc, respectively, for the 35 dose bins.

A commercial 3D image‐based HDR BT TPS could produce DVHs consistent with another independently acquired TPS.

**Figure A1:**
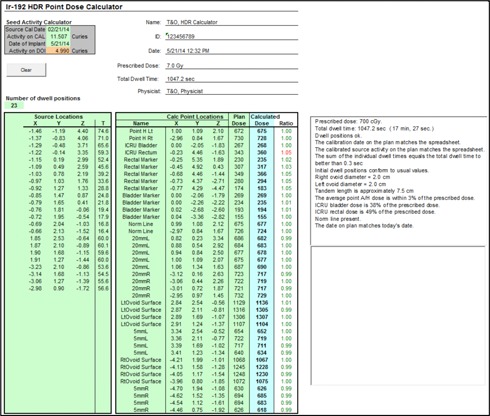
In‐house HDRCalculator software for independent HDR dose calculation and HDR plan quality verification compared to the TPS plan for T&O HDR case. The independently calculated dose on ICRU rectum point indicates 5% off with red color due to its position at anisotropic region.

### E. Volume optimization

#### E.1 Optimization algorithm changes in TPS from line optimization only to line and volume optimization

The three parameters of the upgraded 6.5 version plan were normalized against those already established in the plan created in version 6.1 of the software (Table 1). The average value of the absolute difference (difference range) as a percentage was 1.4% (from −1% to +3%) for TRAK, 6.1% (from −14% to +13%) for ${\text{VOL}}_{100\%\text{Rx}}$, and 1.1% (from −6% to +1%) for Point A dose, respectively (Table 1). The upgraded version 6.5 plans were comparable to the version 6.1 plans with respect to TRAK and Point A dose values. However, the differences in the ${\text{VOL}}_{100\%\text{Rx}}$ values in the two plan optimizations were observed as greater than 10% for two patients. The findings were discussed with physicians before the upgraded TPS version was clinically released. It was learned that a considerable change between the two optimization algorithms could occur. It is imperative that whenever inverse planning optimization tools are upgraded, their impact must be thoroughly validated before clinical use.

#### E.2 Volume optimization consistency between versions

For Case A, the average absolute dwell time difference was 0.8 seconds (2%) with a range of −3.6% to +3.6% between both versions. Source activity was identical because the same source was used. The TRAK difference was 0.2% and the total treatment time difference was only 0.9 s (0.2%). For Case B, the difference between both versions was similar to Case A, with the average absolute dwell time difference being 0.64 s (1.6%) with a range of−1.5% to 3.8%, a 0.3% of TRAK difference, and 2.8 s (0.3%) of total treatment time difference.

Additional optimization (2^nd^ Opt) was performed to verify the reproducibility of volume optimization for Case B, which has a relatively large number of dwell positions (18). Compared to the initial optimization, dwell time from the second optimization differed by only 0.1 seconds for the 3 dwell positions out of 18. The volume optimization could result in a reproducible clinical treatment plan with a total treatment time difference of 0.1 seconds between the two repeated optimizations.

#### E.3 Optimization sensitivity to different optimization time

Table 3 compares the four parameters mentioned above for the five different optimization time scenarios and reports their difference relative to those in the 2‐min optimization time case. The average absolute difference of dwell times (range of difference) was decreased from 2.5% (from −6.2% to 6.0%), 0.7% (from −2.6% to 1.1%), 0.2% (from −0.6% to 0.3%), to 0.1% (from −0.6% to 0.3%) as the optimization time was increased from 10, through to 90 s. The data reveal that the optimizer should run for at least 1 min in order to produce a clinically robust and reproducible treatment plan.

**Table 3:** Optimization sensitivity to optimization times for 10 s, 30 s, 1 min, and 1.5 min relative to the reference (Ref) optimization time of 2 min. Four parameters are compared: each dwell time, source activity, total air Kerma strength, and total treatment time. % difference is defined as: $({\text{Value}}_{\text{ref}}-\text{Value})/{\text{Value}}_{\text{ref}}\times 100$)

		*Volume optimization time*								
*Dwell Position (cm)*		*10 s*	*30s*	*1 min*	*1.5 min*	*2 min (Ref)*	*10 s % Diff*	*30 s % Diff*	*1 min % Diff*	*1.5 min % Diff*
Tandem	119	63.7	64.3	63.9	63.8	63.8	0.2%	−0.8%	−0.2%	0.0%
	118.5	62	63.1	62.6	62.6	62.6	1.0%	−0.8%	0.0%	0.0%
	118	59.8	60.7	60.9	60.9	61	2.0%	0.5%	0.2%	0.2%
	117.5	57.1	58.8	59.1	59.1	59.1	3.4%	0.5%	0.0%	0.0%
	117	54.7	57	57.3	57.3	57.4	4.7%	0.7%	0.2%	0.2%
	116.5	53.2	55.2	55.7	55.8	55.8	4.7%	1.1%	0.2%	0.0%
	116	53.7	54.4	54.5	54.6	54.6	1.6%	0.4%	0.2%	0.0%
	115.5	54.6	53.8	53.7	53.8	53.8	−1.5%	0.0%	0.2%	0.0%
	115	55.8	53.8	53.4	53.4	53.4	−4.5%	−0.7%	0.0%	0.0%
	114.5	56.7	53.4	53.5	53.5	53.4	−6.2%	0.0%	−0.2%	−0.2%
Right Ovoid	120	34.6	35.5	34.7	34.6	34.6	0.0%	−2.6%	−0.3%	0.0%
	119.5	34.3	35.1	34.8	34.8	34.7	1.2%	−1.2%	−0.3%	−0.3%
	119	35	35.1	35	35	34.9	−0.3%	−0.6%	−0.3%	−0.3%
	118.5	35.7	35.1	35.2	35.2	35	−2.0%	−0.3%	−0.6%	−0.6%
Left Ovoid	120	32.7	32.8	32.8	32.8	32.8	0.3%	0.0%	0.0%	0.0%
	119.5	32.5	32.9	32.9	32.9	33	1.5%	0.3%	0.3%	0.3%
	119	31.9	32.8	33.1	33.1	33.1	3.6%	0.9%	0.0%	0.0%
	118.5	31.3	33.1	33.2	33.2	33.3	6.0%	0.6%	0.3%	0.3%
*Source Treatment Activity (mCi)*										
*10 s*	*30 s*	*1 min*		*1.5 min*	*2 min (Ref)*	*% Diff*		*% Diff*	*% Diff*	*% Diff*
4670.6	4670.6	4670.6		4670.6	4670.6	0.0%		0.0%	0.0%	0.0%
*Total Reference Air KERMA [TRAK] (cGy&mdot;cm^2^)*										
*10 s*	*30 s*	*1 min*		*1.5 min*	*2 min (Ref)*	*% Diff*		*% Diff*	*% Diff*	*% Diff*
4388.3	4428.0	4424.9		4425.4	4425.9	0.9%		0.0%	0.0%	0.0%
*Total Treatment Time (s)*										
*10 s*	*30 s*	*1 min*		*1.5 min*	*2 min (Ref)*	*% Diff*		*% Diff*	*% Diff*	*% Diff*
839.3	846.9	846.3		846.4	846.5	0.9%	0.0%		0.0%	0.0%

#### E.4 Comparison of three different volume optimization techniques

The comparison data of three different volume optimization techniques are summarized in Table 4 and a dose distribution comparison for case 1 is shown in Fig. 3. If the deviation from the DVH parameters of a conventional Point A plan results in an improved plan, the cell in Table 4 is colored blue. If the deviation of DVHs results in a worse plan, the cell in Table 4 is colored red. Among the 32 overall dosimetric deviations (4 per case times 8 cases) for each optimization technique, the number of positive deviations (blue in Table 4) occurred 11 (graphical optimization), 9 (inverse planning), and 12 (hybrid optimization) times, while the number of negative deviations (red in Table 4) occurred 3 (graphical optimization) and 6 (inverse planning) times and once for hybrid optimization. When a nonparametric statistical test is used (Mann‐Whitney rank sum test), the plan quality change between positive and negative deviation groups can be compared for each optimization technique. Graphical optimization techniques could produce a dosimetrically better plan than the conventional Point A plan (6.8±4.7% vs. 2.0±1.5% with p‐value of 0.0385). However, the difference was statistically insignificant when using the inverse planning technique (11.6±8.6% vs. 9.8±10.9% with p‐value of 0.2991). With respect to the hybrid inverse planning technique, the p‐value could not be used as there was only one case of a negative deviation (1.5%). The positive deviation value (7.3±5.8%) was much larger than the negative deviation value, suggesting that hybrid inverse planning can produce a clinically superior plan to a conventional Point A plan.

**Table 4:** Percent deviation of DVH parameters for the plans obtained with graphical, pure inverse, and hybrid inverse optimizations from a conventional Point A plan. Compared to conventional Point A plan, cells in blue color represent better plan while cells in red color present worse plan with respect to either target coverage or OARs sparing. Hence, blue cell means higher dose for the target and lower dose for OARs

	*HR‐CTV Vol (cc)*	*Graphical*				*Pure Inverse*				*Hybrid Inverse*			
*Case*		*HR‐CTV D90*	*Rectum D2cc* ^a^	*Bladder D2cc* ^a^	*Sigmoid D2cc* ^a^	*HR‐CTV D90*	*Rectum D2cc* ^a^	*Bladder D2cc* ^a^	*Sigmoid D2cc* ^a^	*HR‐CTV D90*	*Rectum D2cc* ^a^	*Bladder D2cc* ^a^	*Sigmoid D2cc* ^a^
1	37.8	4.7% (3.7 Gy)	0.6% (0.5 Gy)	0%	0%	7.8% (6.1Gy)	2.7% (2.0Gy)	0%	0%	3.9% (3.1Gy)	0%	0%	0%
2	40.6	5.8% (4.1 Gy)	3.6% (2.7 Gy)	0%	0%	4.3% (3.1Gy)	2.9% (2.2Gy)	0%	0%	3.4% (2.4Gy)	1.5% (1.1Gy)	0%	0%
3	37.2	−1.8% (−1.4 Gy)	−4.6% (−3.5 Gy)	0%	−5.5% (−4.3 Gy)	6.5% (4.9Gy)	2.5% (1.9Gy)	7.7% (6.9Gy)	−9.7% (−7.6 Gy)	2.4% (1.8Gy)	−2.3% (−1.7 Gy)	0%	−11.9% (−9.3 Gy)
4	10.2	0%	−11.0% (−9.3 Gy)	0%	0%	0%	−16.1% (−13.6 Gy)	0%	0%	0%	−16.1% (−13.6 Gy)	0%	0%
5	3.9	0%	0%	0%	−18.2% (−14.5 Gy)	0%	0%	0%	−30.8% (−24.5 Gy)	0%	0%	0%	−19.6% (−15.6 Gy)
6	28.7	0%	0%	−5.4% (−5.5 Gy)	0%	0%	0%	5.7% (5.8Gy)	0%	0%	0%	−7.8% (−7.9 Gy)	0%
7	99.7	2.9% (2.0 Gy)	−1.7% (−1.3 Gy)	0%	0%	5.2% (3.6Gy)	14.3% (11.0Gy)	32.6% (24.8Gy)	0%	3.4% (2.4Gy)	−4.6% (−3.5 Gy)	0%	0%
8	57.5	4.6% (3.6 Gy)	0%	0%	−10.3% (−8.4 Gy)	6.7% (5.2Gy)	0%	0%	−17.7% (−14.4 Gy)	2.9% (2.3Gy)	0%	0%	−9.0% (−7.4 Gy)

a ^a^ Rectum, Bladder, and Sigmoid D2cc= dose to 2cc volume of rectum, bladder, and sigmoid, respectively.

HR‐CTV D90= minimum dose to cover 90% of high risk CTV volume.

As the dose distribution is manually adjusted for the graphical optimization and hybrid inverse techniques, a planner can modify and evaluate the dose distribution in real time. This gives the graphical optimization and hybrid inverse planning techniques an advantage over the inverse planning technique in this respect. Although the pure inverse planning technique is able to improve target dose in five out of eight cases (5/8) with a magnitude increase in HR‐CTV D90 much higher than compared to the graphical optimization technique (6.1±1.4% vs. 3.2±0.6%), it also increased OARs doses. We observed elevated doses to OARs in 4/8 and 3/8 cases for rectum and bladder, respectively, with a dose increase of 9.8±10.9%, reaching as high as 14.3% for the rectal dose and 32.6% for the bladder dose.

Pure inverse planning can result in a significantly different isodose line distribution depending on HR‐CTV (see Fig. 3(b)). Due to the need for manual adjustment when using the graphical optimization and hybrid inverse optimization techniques, they take almost twice as much as time to generate as compared to a conventional Point A plan (Fig. 3(e)). Despite iterative optimization processes by adjusting dose constraints and evaluating DVHs, pure inverse optimization takes the least optimization time (9.5±2.1min).

### F. End‐to‐end test

An end‐to‐end test was performed on a HDR QA phantom to ensure the correct operation of the procedure from 3D image acquisition, planning and delivery for 3D CT‐ or MRI‐guided HDR treatment. During the end‐to‐end tests, printing functionality of the TPS was also tested to ensure that the TPS could print both hard copies (paper) and electronic copies to PDF (portable document format). In addition, data transfer from an updated TPS to the afterloader unit was manually verified by comparing their hardcopies. All dwell times, corresponding dwell positions, treatment date, and source activity between the TPS and the afterloader unit were verified. Full nondosimetric image‐related tests are listed in Appendix B. The authors recommend imaging geometry consistency and accuracy for CT or MR have less than 1 mm uncertainty (see Appendix B).

## IV. DISCUSSION

Many institutions just follow the vendor's test list that refers to 2D image‐based guidelines based on AAPM TG 53 and TG 56. The commercial TPS commissioning or upgrade validation procedures provided by a vendor are intended to verify system configuration and to test each component of the TPS. These components include the definition of the treatment unit, installation of source and image geometry, and configuration of the image import methods such as DICOM image transfer. The purpose of the system verification test is, for example, to check the functionality of reference point entry, printer, isodose display, dose calculation, treatment plan, and image transfer. In order to upgrade a commercial TPS to a version capable of performing 3D image‐based HDR treatments, additional commissioning procedures were added to the cohort of vendor‐recommended procedures for validation purposes: applicator reconstruction accuracy, imaging tools in image‐based TPS, ^192^Ir source model and source data, dose calculation algorithm (TG 43 formalism) for three benchmark cases, DVH, volume optimization algorithm in 3D image‐based planning, and end‐to‐end test from imaging, planning to delivery.

In this study, we presented the tests and results, addressing the 3D‐image based HDR BT planning issues related to the applicator library and titanium applicator reconstruction accuracy validations as well as different volumetric optimization techniques in 3D HDR BT planning. A virtual applicator library for applicator reconstruction with 3D image has been available for most HDR BT TPSs. It may be erroneously assumed that a virtual applicator (Figs. 1(c) and (f)) matches the corresponding physical applicator. However, we found that the right ovoids of titanium FSD T&O set (Varian Medical Systems, Inc.) did not match with the corresponding ovoids in their virtual applicator libraries. For example the right side of the virtual library in a TPS is defined as a patient's right as they lie in the supine position, while the right side in a physical applicator set is defined as a physician's right. As the physician prepares for the insertion of the T&O their right is analogous to the patients left. In the case of the mini ovoid with a 1.6 cm diameter which has a medial flat side, the confusion of the correct location of right or left ovoid can cause considerable dosimetric errors. The dimensions of the applicator in the virtual applicator library should be also validated with their nominal values before clinically using them. In another example, a titanium FSD T&O applicator was recently replaced with a click‐fit FSD T&O (Varian Medical Systems, Inc.) with a 0.95±0.1 mm tip thickness while the old FSD T&O has a tip thickness of 0.6±0.1 mm. However the dimensions in the applicator library (BrachyVision version 10.0) were not updated accordingly. It turned out that the difference between nominal and applicator library values was less than 0.5 mm. As the tip thickness affects applicator reconstruction accuracy, it is important for a medical physicist to validate each applicator library model clinically used and compare it with its nominal value and its measured value on 3D images. For applicator library modeling inaccuracies greater than 1 mm, it is recommended to report these to the vendor and to consider accounting for them during treatment planning. During the initial TPS commissioning or during later updating, methodical procedures must be followed that include reporting unexpected variation or errors to the vendor and modifying workflow accordingly. We found higher variations when inverse optimization running time was less than a minute, so all inverse optimization in our clinic runs for at least 1 min.

When MR image datasets are used exclusively without additional CT datasets for HDR planning, the reconstruction accuracy of the MR imaged titanium T&O applicators is poor due to the artifacts and distortions inherent to this particular modality.^(23)^ Clinical validation of titanium T&O including safety tests and artifact and distortion assessments are performed before clinical use.^23^, ^39^ GEC‐ESTRO recommends the use of a MRI‐marker catheter when a plastic applicator is reconstructed on MR images without the use of secondary CT or X‐ray.^(5)^ Previous studies have reported on applicator reconstruction with the use of MRI‐marker catheters and associated applicator library when a plastic applicator is reconstructed on MRI only.^40^, ^41^ Haack et al.^(42)^ presented applicator reconstruction accuracy for a plastic tandem‐and‐ring applicator using phantom and patient‐data studies. The GYN GEC‐ESTRO working group provided recommendations for the commissioning and applicator reconstruction of 3D BT planning, but the report lacked details describing titanium applicator commissioning and reconstruction. Titanium applicator reconstruction accuracy validation studies on MRI are still lacking.^5^, ^42^, ^43^ Haack et al.^(42)^ presented the pioneering results on testing titanium ring applicators by using phantom and *in vivo* datasets. They recommended using titanium susceptibility artifacts as an applicator reconstruction landmark, but the susceptibility artifacts are inherently uncertain in terms of its location and magnitude due to its very nature. No quantitative analysis on titanium applicator reconstruction accuracy was presented. The recommendation on titanium applicator reconstruction was presented in the GEC‐ESTRO working group report.^(5)^ To our best knowledge, no report presenting quantitative comparisons of the reconstruction accuracy of titanium applicators over that of CT or X‐ray has been published up to this point.

We presented the validation of titanium T&O reconstruction accuracy on high resolution (3 T) MRI compared to conventional 2D X‐ray imaging. Ideally, CT is the best choice for an imaging modality to establish a set of reference image datasets to assess titanium T&O reconstruction accuracy on MRI. However, the cost of these additional CT scans will not be reimbursed if MRI datasets are used for planning. While X‐rays are used to determine titanium T&O applicator displacement during patient transfer, X‐rays are used as reference datasets in this study. The artifacts generated from titanium applicator end‐tips present a particular set of challenges when imaged on MRI and were first investigated^(23)^ when a novel marker‐flange was proposed to improve reconstruction accuracy when a titanium applicator was used in conjunction with a virtual library.^(44)^ A titanium applicator requires additional care even when using CT‐based planning, since the bright signal of the titanium tip of the tandem does not match the location of the physical tip. Due to density difference between titanium and soft tissue, the TPS visualizes the smooth‐filtered bright signals as a titanium applicator. It is recommended to define the tip of titanium applicator on CT by checking the Hounsfield unit change not by the bright titanium signal end.

3D BT image guidance provides the opportunity to use a 3D volume‐based dwell time optimization instead of line‐based one. ABS guidelines^(13)^ define the term optimization as “the sophisticated process of achieving certain dose values at points or volumes within the implant”. A number of dosimetric and clinical reports compare conventional Point A plans to 3D volume optimized plans.^32^, ^45^, ^46^, ^47^ Inverse optimization approaches for 3D image‐guided, GYN HDR have been described;^45^, ^48^, ^49^, ^50^ however, studies testing different volume optimization approaches in terms of dosimetry and optimization time are still lacking. We compared the dosimetric and optimization‐time of three different volume optimization approaches: inverse optimization, graphical optimization, and the hybrid inverse optimization technique. As a first feasibility study of the hybrid inverse optimization technique, the first implant plans of eight patients were analyzed and introduced in this study as a part of a commissioning procedure for our 3D image guided volumetric GYN HDR program. Regarding the hybrid inverse optimization technique, studies using a larger number of plans will be prepared for publication. We recommend that each clinic, however, test dosimetry and additional optimization times for currently available volume optimization techniques by using at least minimal number of plans before each institutional method is decided. These may include manual dwell time changes, graphical optimization, or hybrid inverse optimization. In this study, pure inverse planning takes the least optimization time but can result in a significantly different isodose line distribution depending on HR‐CTV (see Fig. 3). ABS guidelines^(13)^ and EMBRACE (A international study on MRI‐guided brachytherapy in locally advanced cervical cancer) protocols do not recommend the exclusive use of inverse planning to select a source loading as this may result in substantial or undesirable changes to the treatment plan. Inverse optimization does not take into account the high dose volumes within the high‐risk (HR) CTV and noncontoured OAR such as the vagina, ureter, vessels, nerves or connective tissue in the pelvis. Large, undesirable cold regions in the target area or hot regions in noncontoured OAR may occur which may precipitate adverse clinical events. When 3D image guidance is used, it is recommended that the volume optimization be started from a conventional Point A‐based plan. The advantage of the hybrid inverse optimization technique is to produce plans that increase the HR‐CTV while sparing the OARs due to the real‐time DVH adjustment that this plan affords.

We used our in‐house software, HDRCalculator, to independently check doses in the HDR plans. In addition to a second physicist's check, the HDRCalculator also independently checks plan integrity by using coordinate information of the points in a HDR plan. However, the majority of clinics use commercial software, such as BrachyCheck (Oncology Data Systems, Inc., Oklahoma City, OK), to independently calculate dose to meet regulatory and billing requirements. For clinics using commercial software, it is recommended that checklists are used for the planner and the 2nd check physicist as proposed in the safety checklist of medical physics practice guidelines, for an example of a checklist for HDR breast brachytherapy (see Fig. B.3 in Fong et al^(51)^).

We validated the dosimetric stability when an algorithm change occurred, for example from a line‐optimization to a volume optimization when performing BT planning using the TPS. Similarly, new 3D image‐based TPS inverse‐planning routines should also be verified before being clinically used. Its ability to produce a clinically optimal dose distribution should be evaluated and compared to other optimization methods for any HDR applicator in all clinical sites. These test plans have to include the variety of possible clinical scenarios; for example, all clinically available cylinder sizes and various treatment lengths for the clinical GYN VC cases. Based on these clinical test cases, a set of dose constraints (a class solution) could be established for dose‐volume objectives and weighting factors on target, OARs, any virtual lines, and volumes.

## V. CONCLUSIONS

To clinically implement a 3D image‐based HDR program for cervical cancer we provided details for the initial commissioning or subsequent updating of a 3D image‐based TPS, including virtual applicator library validations, applicator reconstruction accuracy tests on high resolution (3T) MRI, DVH, and volume optimization validation. In particular, comprehensive procedures to commission volume optimization processes in 3D image‐based planning were presented for the difference between line and volume optimizations, volume optimization consistency between versions and its sensitivity to different optimization times. For clinical GYN T&O cases, different volumetric optimization techniques were compared. End‐to‐end testing was performed using a HDR QA phantom to ensure no errors or inconsistencies occurred from 3D imaging acquisition through to planning and delivery. By following the proposed commissioning procedures, a clinically safe implementation of a 3D image‐based TPS can be achieved for the treatment of cervical cancer using HDR BT.

## COPYRIGHT

This work is licensed under a Creative Commons Attribution 4.0 International License.

## APPENDICES

### Appendix A: In‐house HDRCalculator Software

### Appendix B: Summary of Tests for 3d Image‐based hdr Commissioning

**Table A1:** Summary of tests for commissioning of 3D image‐based HDR brachytherapy TPS for VC and T&O cases of gynecological cancer treatment

*Test*	*Tolerance*	*Pass/Fail*	*Reference*
*Dosimetric [D]*			
D1 Remote after loading unit (RAU) setup	Properly setup		(20)
D2 HDR source setup	Properly setup		(20)
D2.1 TG43 parameters check	Properly setup		(28)
D2.2 TG43 calculations	<±2%		(15)
D3 Isodose display test & setup	Functional		
D4 3D dose display & DVH test	Functional		(20)
D5 T&O dose calculation: consistency	<±2%		(15)
D5.1 Dwell times (total & each dwell position)	<±2%		(15)
D5.2 TRAK (Total air KERMA strength)	<±2%		(15)
D5.3 Dose calculation reproducibility	<±2%		(15)
D5.4 DVH comparison	<±2%		(20)
D6 VC dose calculation: consistency	<±2%		(15)
D6.1 Dwell times (total & each dwell position)	<±2%		(15)
D6.2 TRAK (Total air KERMA strength)	<±2%		(15)
D6.3 Dose calculation reproducibility	<±2%		(15)
D7 Independent dose calculation consistency			
(’HDRCalculator’ as in‐house software)	<±2%		(15)
*Nondosimetric [N]*			
N1 Configure DICOM image transfer	Functional		(20)
N2 Imaging import (C‐arm / CT / MRI)	Functional		(20,5)
N3 CT/MR imaging geometry consistency	<1 mm		
N4 CT import check	Functional		(20,5)
N5 Digitizer test	<1 mm		
N6 Creating a plan with CT or MRI	Functional		(20,5)
N7 Applicator library template: accuracy test	Functional		(5)
N8 Reference point entry test	Functional		(20)
N9 Printer test	Functional		(20)
N10 Genetic‐text print format (clinical print & DVH)	Functional		
N11 Print setup: PDF	Functional		
N12 Plan in TPS: unlock and lock check	Functional		
N13 User login setup	Functional		
N14 Current date & time check	Functional		
N15 T&O and VC plan template check	Functional		
N16 T&O and VC optimization template check			
*End‐to‐end TEST: Imaging to Delivery [E]*			(20)
E1 VC			
E2 CT based T&O planning and delivery			(5)
E3 MRI based T&O planning and delivery			(5)
